# Anti-PD-1 combined sorafenib versus anti-PD-1 alone in the treatment of advanced hepatocellular cell carcinoma: a propensity score-matching study

**DOI:** 10.1186/s12885-022-09173-4

**Published:** 2022-01-11

**Authors:** San-Chi Chen, Yi-Hsiang Huang, Ming-Huang Chen, Yi-Ping Hung, Rheun-Chuan Lee, Yu-Yun Shao, Yee Chao

**Affiliations:** 1 Institute of Clinical Medicine, National Yang Ming Chiao Tung University, Taipei, Taiwan; 2 Faculty of Medicine, National Yang Ming Chiao Tung University, Taipei, Taiwan; 3 Division of Medical Oncology, Center for Immuno-oncology, Department of Oncology, Taipei Veterans General Hospital, No. 201, Sec. 2, Shipai Road, Taiwan 11217 Taipei, Taiwan; 4 Division of Gastroenterology and Hepatology, Department of Medicine, Taipei Veterans General Hospital, Taipei, Taiwan; 5 Department of Radiology, Taipei Veterans General Hospital, Taipei, Taiwan; 6 Department of Oncology, National Taiwan University Hospital, Taipei, Taiwan; 7 Graduate Institute of Oncology, National Taiwan University College of Medicine, Taipei, Taiwan; 8 Department of Medical Oncology, National Taiwan University Cancer Center, Taipei, Taiwan

**Keywords:** Hepatocellular carcinoma (HCC), Anti-PD-1, Nivolumab, Pembrolizumab, Sorafenib, Multiple-kinase inhibitor, Vascular endothelial growth factor (VEGF), Combination therapy, Propensity score matching (PSM)

## Abstract

**Background:**

Vascular endothelial growth factor (VEGF) plays a role in the tumor microenvironment. Sorafenib, which inhibits the VEGF pathway, has an immune-modulation function but lacks substantial clinical data. This study aims to explore the efficacy of anti-PD-1 combined sorafenib in advanced hepatocellular carcinoma (HCC).

**Methods:**

HCC patients who underwent anti-PD-1 treatment at Taipei Veterans General Hospital (Taipei, Taiwan) between January 2016 and February 2019 were reviewed. The efficacy was compared between groups after propensity-score matching.

**Results:**

There were 173 HCC patients receiving anti-PD-1. After excluding unsuitable cases, 140 patients were analyzed, of which 58 received combination therapy and 82 received anti-PD-1 alone. The combination therapy had a trend of higher CR rate (8.6% vs. 4.9%, ns.), ORR (22.4% vs. 19.5%, ns.) and significantly higher DCR (69.0% vs. 37.8%, *p* < 0.05) comparing to anti-PD-1 alone. After matching, combination group achieved longer progression-free survival (3.87 vs. 2.43 months, *p <* 0.05) and overall survival (not reached vs. 7.17 months, *p <* 0.05) than anti-PD-1 alone, without higher grade 3/4 AE (10.3% vs. 7.1%, *p =* 0.73). The tumor response varied among different metastatic sites, with high responses in adrenal glands, peritoneum and lungs. The more AFP declined (> 10, > 50 and > 66%), the higher the ORR (70, 80 and 92%) and CR rates (30, 35 and 58%) were achieved at day 28.

**Conclusions:**

This is the first study to demonstrate the combination of anti-PD-1 and sorafenib had better efficacy and survival benefit. A prospective randomized study is needed to confirm this finding.

**Supplementary Information:**

The online version contains supplementary material available at 10.1186/s12885-022-09173-4.

## Background

HCC is the second most common cause of cancer-related death in Taiwan [1] and ranks fourth worldwide [2]. In advanced HCC, the prognosis is poor and treatment options are limited. In recent years, anti-PD-1 has become the standard treatment for many types of cancer, including HCC. However, nivolumab failed to prove its survival benefit over sorafenib in 1st line treatment [3]. In the absence of biomarker, to combine anti-PD-1 and other drugs becomes a feasible treatment option. The major potential drugs include anti-vascular endothelial growth factor monoclonal-antibody (anti-VEGF mAb), anti-CTLA-4 and multiple-kinase inhibitors (MKI). Among these, anti-VEGF mAb is the most successful.

Recently, vascular endothelial growth factor (VEGF) was found to play a role in the tumor microenvironment. The blockade of the VEGF pathway increases the infiltration of effector immune cells via normalization of abnormal tumor vasculature [4]. In a phase 1b study, GO30140, the addition of bevacizumab to atezolizumab demonstrated longer PFS than atezolizumab alone, which made this combination a promising treatment option for HCC [5]. Thereafter, atezolizumab combined bevacizumab resulted in better PFS and OS outcomes than sorafenib in Imbrave150 [6]. Based on these data, this regimen become the first recommended combination therapy in the first line setting of advanced HCC.

Sorafenib, a multiple kinase inhibitor that inhibits VEGF pathway, has been the first-choice drug recommendation in advanced HCC for a decade [7]. The inhibition of VEGF pathway by sorafenib not only enhances functions of effector T cells in tumor microenvironment [8], but also decreases suppressive immune cells [8–10]. Besides, sorafenib-treated HCC tissue significantly increased PD-L1 expression in immune cells [11]. When combined with anti-PD-1, sorafenib inhibited tumor growth by inducing effective natural killer cells [12]. These data suggested that sorafenib may be a potential candidate to combine with anti-PD-1.

There was only one case report showing that the combination of anti-PD-1 and sorafenib achieved complete response with advanced HCC [13]. However, there is no study providing survival benefit with the combination of multiple-kinase inhibitor and anti-PD-1. The aim of this study is to explore the clinical benefit of the addition of sorafenib to anti-PD-1 comparing with anti-PD-1 alone.

## Methods

### Patients and study design

Between January 2016 and February 2019, 173 HCC patients who underwent anti-PD-1 treatment with or without sorafenib at Taipei Veterans General Hospital (Taipei, Taiwan) were retrospectively reviewed. Patients with Child-Pugh Score C or those without efficacy assessment were excluded, therefore, 140 patients were enrolled for this study. Information regarding patient characteristics, including patient age, sex, history of viral hepatitis, liver function, tumor markers, tumor stage, and tumor treatment history was collected and analyzed. The diagnosis of HCC was confirmed histologically or clinically based on the American Association for the Study of Liver Diseases criteria.

Liver function was established by Child-Pugh score and ALBI grade. Cancer staging used the Barcelona clinic liver cancer (BCLC) staging system. The up-to-11 criteria combined the number of tumors and size of the largest one, with the sum being no more than 11 was applied for tumor burden. This study has been approved by the institutional review board of Taipei Veterans General Hospital, which is the appropriate regulatory agency to review research on both adults and children. (VGHTPE-IRB: 2017–10-005 BC). This work was supported by grants from the Ministry of Health and Welfare and the Center of Excellence for Cancer Research (MOHW110-TDU-B-211-144,019) and Taipei Veterans General Hospital (V107B-036 to S-CC).

### Outcome assessment

Treatment responses were assessed by Computed Tomography scan or Magnetic Resonance Imaging every 2–3 months in accordance with RECIST and mRECIST criteria (19). Immune-related adverse events were graded according to the National Cancer Institute Common Terminology Criteria for Adverse Events version 4.0. Progression-free survival (PFS) was defined as the time period from the beginning of treatment until disease progression or death, time to response (ToR) from the beginning of treatment until documentation of tumor response, duration of response (DoR) from documentation of tumor response to disease progression, and overall survival (OS) from the beginning of treatment to death.

### Statistical analysis

To reduce confounding, propensity-score was used to match patients treated with anti-PD-1 plus sorafenib to those treated with anti-PD-1 alone. Variables including age, sex, etiology, cirrhosis, Child-Pugh score, portal vein thrombosis (PVT), metastasis, AFP level and sorafenib experienced were used for matching. Inverse probability of treatment weighting (IPTW) was used to confirm the analysis. For each patient, propensity score was calculated with logistic regression model using baseline characteristics, including age, sex, etiologies, cirrhosis, liver function, portal vein thrombosis, metastasis, AFP > 400, sorafenib experienced, and ECOG, tumor burden as well. Generalized estimating equation was used to compare efficacy between groups.

Student’s t-test was used to compare continuous variables and Chi-square test or Fisher’s exact test to categorical variables between groups. Cox-regression analysis was used to determine risk for disease progression and mortality and the Log-rank test to compare Kaplan-Meier curves. Propensity-score matching was done with caliper width of 0.1. SPSS version 24.0 was used for the statistical analysis (IBM Corp. Released 2016. IBM SPSS Statistics for Windows, Version 24.0. Armonk, NY: IBM Corp.). *p* < 0.05 was considered to indicate a statistically significant difference.

## Results

### Patient characteristics

Among 140 HCC patients, 58 patients had a combination of anti-PD-1 and sorafenib and 82 had anti-PD-1 alone, with the median duration of follow-up being 9.1 months. The mean of sorafenib dose in combination group was 351 ± 168 mg. Nivolumab was prescribed for 123 (87.9%) patients and pembrolizumab for 17 (12.1%). Before matching, the combination group had significantly higher ALBI grade, more PVT (74.1% vs. 46.3%, *p* < 0.05) and more advanced BCLC stage (stage C 94.8% vs. 81.7%, *p* < 0.05). After matching, clinical variables were not different between groups (Table 1).

**Table 1:** Demographic and clinical characteristics

	Before matching					After matching				
Characteristic	Anti-PD-1 plus Sorafenib (*n* = 58)		Anti-PD-1 alone (*n* = 82)		*p* value	Anti-PD-1 plus sorafenib (*n* = 58)		Anti-PD-1 alone (*n* = 42)		*p* value
Age (mean ± SD)	69.1 ± 13.4		61.7 ± 12.3		0.25	69.1 ± 13.4		61.2 ± 12.7		0.25
Male	45	77.6%	64	78.0%	0.95	45	77.6%	29	69.0%	0.77
Etiology										
HBV	35	60.3%	53	64.6%	0.61	35	60.3%	28	66.7%	0.52
HCV	9	15.5%	15	18.3%	0.67	9	15.5%	8	19.0%	0.64
Alcohol	29	50.0%	36	43.9%	0.48	29	50.0%	18	42.9%	0.48
Cirrhosis	21	36.2%	26	31.7%	0.58	21	36.2%	17	40.5%	0.66
Child-Pugh score										
A	47	81.0%	66	80.5%	0.94	47	81.0%	29	69.0%	0.17
B	11	19.0%	16	19.5%		11	19.0%	13	31.0%	
ALBI grade										
Grade 1	10	17.2%	31	37.8%	< 0.05	10	17.2%	12	28.6%	0.34
Grade 2	41	70.7%	47	57.3%		41	70.7%	27	64.3%	
Grade 3	7	12.1%	4	4.9%		7	12.1%	3	7.1%	
BCLC stage										
B	3	5.2%	15	18.3%	< 0.05	3	5.20%	3	7.1%	0.68
C	55	94.8%	67	81.7%		55	94.8%	39	92.9%	
PVT	43	74.1%	38	46.3%	< 0.05	43	74.1%	29	69.0%	0.58
Metastasis	30	51.7%	49	59.8%	0.35	30	51.7%	26	61.9%	0.31
AFP > 400 (ng/mL)	34	58.6%	43	52.4%	0.47	34	58.6%	29	69.0%	0.29
Sorafenib experienced	37	63.8%	49	59.8%	0.63	37	63.8%	28	66.7%	0.31

*PVT* portal vein thrombosis

### Treatment response

**Fig. 1:**
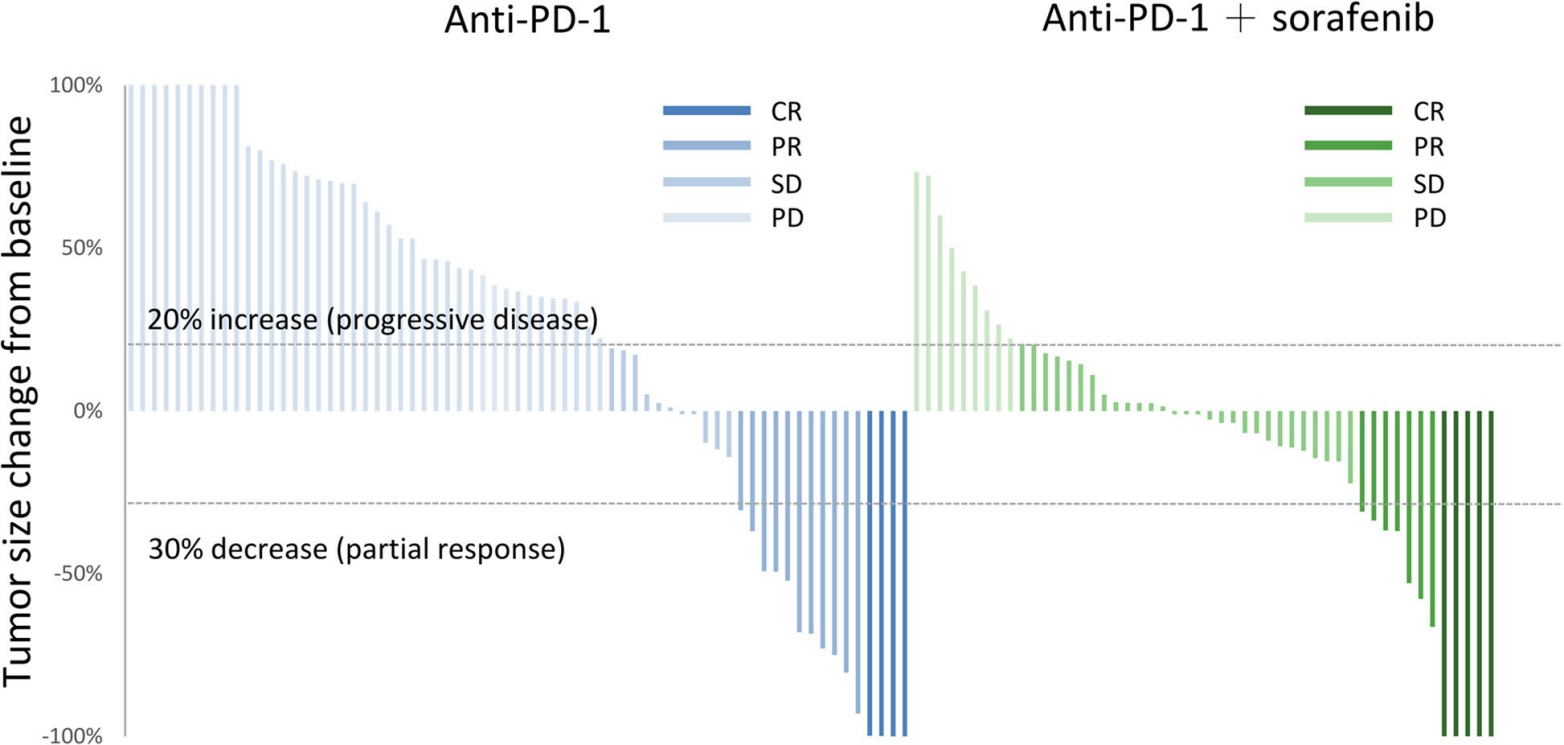
Maximum Change from Baseline in the Sum of Longest Diameters. Anti-PD-1 plus sorafenib demonstrated more tumor shrinkage and disease control. PD was defined as 20% increase in tumor size, while partial response had a 30% decrease. (CR, complete response; PR, partial response; SD, stable disease; PD, progressive disease)

The combination therapy had a trend of higher CR rate (8.6% vs. 4.9%, ns.), ORR (22.4% vs. 19.5%, ns.) and significantly higher DCR (69.0% vs. 37.8%, *p <* 0.05) comparing to anti-PD-1 alone. After matching, the higher DCR remained significant with combination therapy (Table 2). The change of tumor size could be evaluated in 122 patients, which showed combination therapy achieved more tumor shrinkage and better disease control (Fig. 1).

**Table 2:** Treatment response

Before matching	Anti-PD-1 plus sorafenib (*n* = 58)		Anti-PD-1 alone (*n* = 82)		*p* value
ORR	13	22.4%	16	19.5%	0.68
DCR	40	69.0%	31	37.8%	< 0.05
CR	5	8.6%	4	4.9%	
PR	8	13.8%	12	14.6%	
SD	27	46.6%	15	18.3%	
After matching	Anti-PD-1 plus sorafenib (*n* = 58)		Anti-PD-1 alone (*n* = 42)		*p* value
ORR	13	22.4%	9	21.4%	0.91
DCR	40	69.0%	14	33.3%	< 0.05
CR	5	8.6%	3	7.1%	
PR	8	13.8%	6	14.3%	
SD	27	46.6%	5	11.9%	

*CR* complete response, *PR* partial response, *SD* stable disease, *PD* progressive disease, *ORR* objective response rate, *DCR* disease-control rate

### Progression-free survival and overall survival

**Fig. 2:**
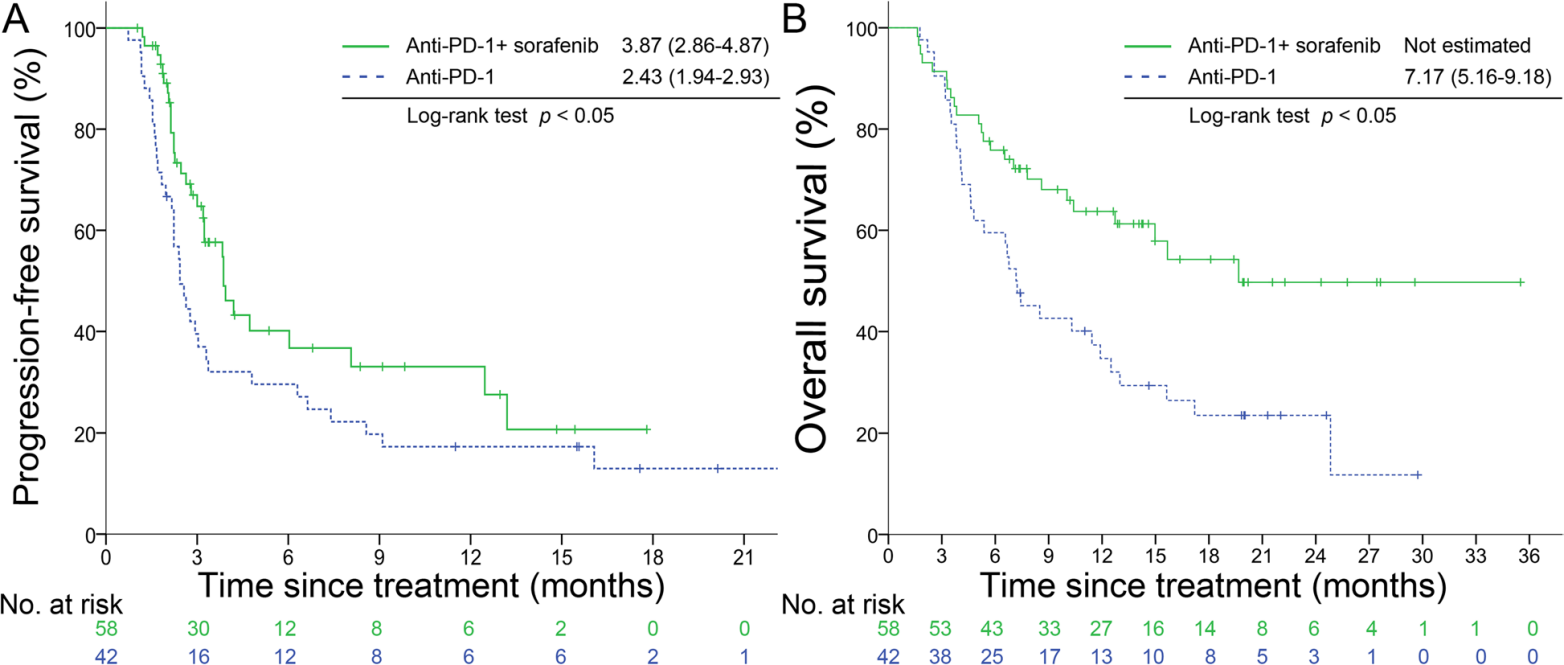
Progression-free survival and overall survival. Kaplan-Meier curves for progression-free survival (**A**) and overall survival (**B**)

**Fig. 3:**
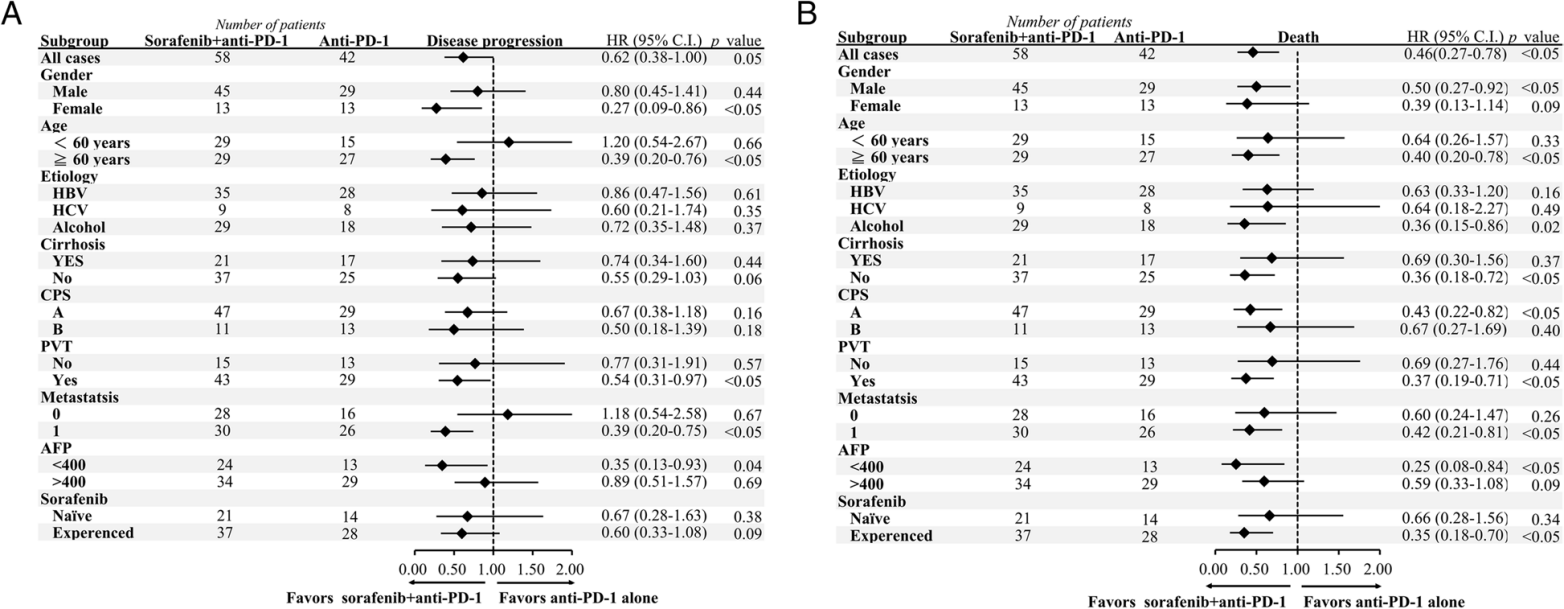
Subgroup analysis in the matched cohort. Subgroup analysis for disease progression (**A**) or death (**B**). PVT denotes portal vein thrombosis

After matching, combination therapy showed longer PFS (3.87 vs. 2.43 months, *p <* 0.05) than anti-PD-1 alone (Fig. 2A). Combination therapy had a lower risk of disease progression (HR 0.62, [0.38–1.00]) and most of the subgroups favored combination therapy except age < 60 years and no metastasis (Fig. 3A). Combination therapy achieved longer OS (not estimated vs. 7.17 months, *p <* 0.05) (Fig. 2B) and lower risk of death (HR 0.46, [0.27–0.78]). Subgroup analysis showed all groups favored combination therapy (Fig. 3B). The time to response were not significantly different with combination therapy and anti-PD-1 alone (2.16 vs. 3.53 months, *p* = 0.20). The duration of response was not different neither (8.89 vs. 8.05 months, *p* = 0.45). After IPTW, the combination group still demonstrated decreasing risk of disease progression (HR 0.18, *p* value < 0.001), and death (HR 0.42, *p* value = 0.03).

### Early AFP response associated with image response

**Fig. 4:**
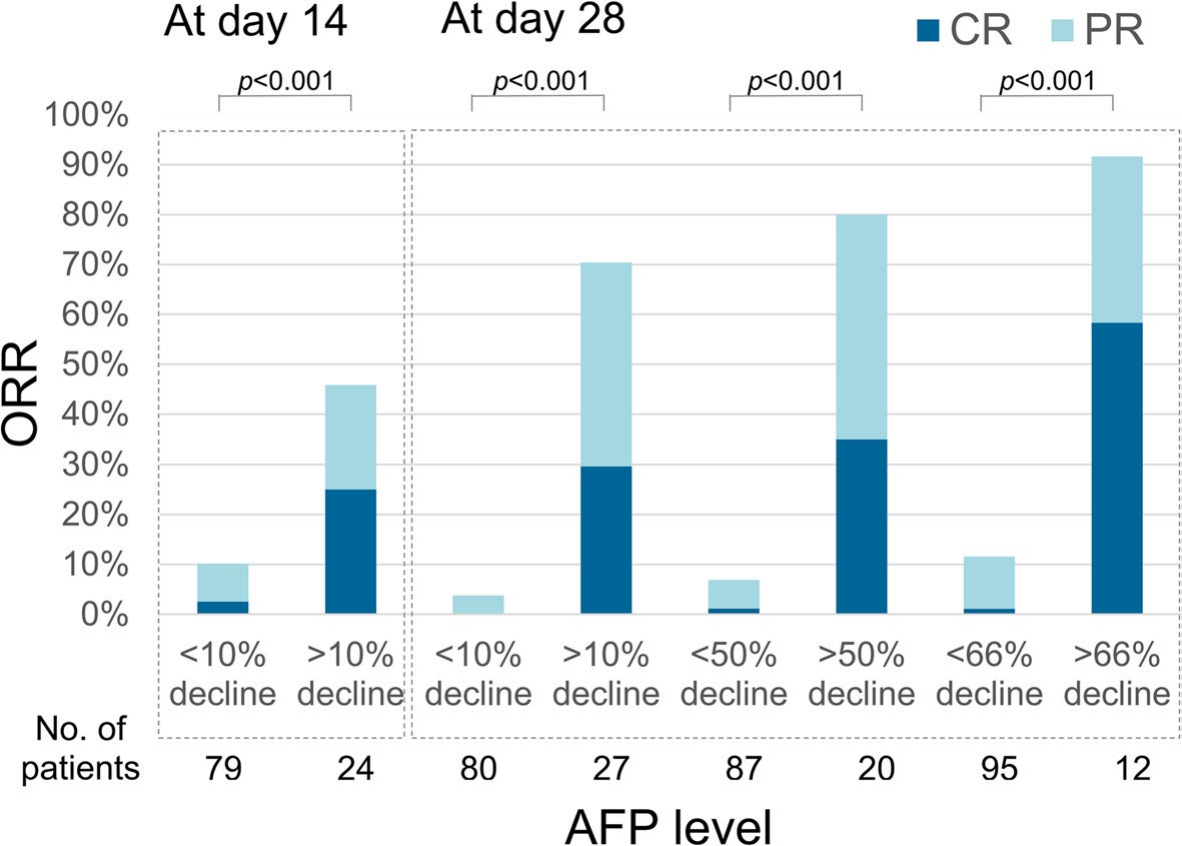
The association between AFP response and image response. AFP response was strongly associated with the subsequent image response, which could be observed as early as 14 days. The more decline of AFP level, the higher ORR and CR rate. (ORR, objective response rate; CR, complete response; PR, partial response)

The early decline of AFP level was strongly associated with the subsequent image response, which could be observed at days14. Patients with AFP level more than 10 (ng/mL) and declined more than 10% from baseline had higher ORR (46% vs. 10%, *p <* 0.001) and CR rate (25% vs. 3%, *p <* 0.001). At day 28, the more AFP declined (> 10, > 50 and > 66%), the higher ORR (70, 80 and 92%) and CR rate (30, 35 and 58%) achieved (Fig. 4).

### Organ-specific tumor response

The tumor response among different metastatic sites was assessed. The ORR was 19.4% in the liver (*n* = 129), 33.3% in lung (*n* = 15), 25.0% in lymph node (*n* = 7), 40.0% in peritoneum (*n* = 5), 0% in bone metastasis (*n* = 5) and 50% in adrenal metastasis (*n* = 4). Tumor thrombosis was regressed in 7.4% of cases (*n* = 81), including one CR and 2 PR in patients with PVT, 2 PR with inferior vena cava thrombosis and one CR with superior mesenteric vein thrombosis (Table 3).

**Table 3:** Organ-specific tumor response (*n* = 140)

Sites	CR		PR		SD		ORR	DCR
Liver (*n* = 129)	9	7.0%	16	12.4%	40	31.0%	19.4%	50.4%
Thrombosis (*n* = 81)	2	2.5%	4	4.9%	25	30.9%	7.4%	38.3%
Lung (*n* = 15)	2	13.3%	3	20.0%	3	20.0%	33.3%	53.3%
Lymph node (*n* = 7)	0	0.0%	1	14.3%	1	14.3%	14.3%	28.6%
Peritoneum (*n* = 5)	1	20.0%	1	20.0%	0	0.0%	40.0%	40.0%
Bone (*n* = 5)	0	0.0%	0	0.0%	1	20.0%	0.0%	20.0%
Adrenal gland (*n* = 4)	0	0.0%	2	50.0%	0	0.0%	50.0%	50.0%

*CR* complete response, *PR* partial response, *SD* stable disease, *PD* progressive disease, *ORR* objective response rate, *DCR* disease control rate

### Toxicity

Combination therapy had more grade 3/4 AE without statistically significant (10.3% vs. 7.1%, *p =* 0.73) (Supplemental Table 1). Generally, the incidence of grade 3/4 AE was less than 5% in each site. Both groups had hepatitis, pneumonitis and skin toxicities. Especially, there was one sick sinus syndrome developed with combination therapy, which has been published as the first case report [14].

## Discussion

The major findings of this study included (1) the combination of anti-PD-1 and sorafenib demonstrated better tumor control, longer PFS and OS comparing with anti-PD-1 alone, (2) combination therapy did not increase grade 3/4 toxicities significantly, and (3) deeper AFP response was a surrogate marker for deeper image response.

In recent years, anti-PD-1 has become a breakthrough treatment for advanced HCC. Comparing with sorafenib, nivolumab demonstrated a higher ORR (15% vs. 7%), but similar DCR (55% vs. 58%), PFS (3.7 vs. 3.8 months) and OS (16.4 vs. 14.7 months) [3]. Therefore, anti-PD-1 alone failed to prove its superiority to sorafenib in the first-line treatment. In the absence of biomarker, combination of anti-PD-1 and other anti-tumor agents becomes a feasible treatment strategy to achieve better efficacy.

In this study, we found the addition of sorafenib to anti-PD-1 greatly increased DCR from 33 to 69%. In addition, the degree of deterioration with combination therapy was mild comparing with anti-PD-1 alone in the waterfall plot. Such efficacy translated into a lower risk of disease progression (HR 0.62) and death (HR 0.46). From past research, including SHARP and Asian-Pacific study, high DCR of sorafenib lowered risk of progression (HR 0.58 and 0.57) and death (HR 0.69 and 0.68) comparing with placebo [7, 15]. These data suggested that sorafenib retains its anti-tumor properties when combined with anti-PD-1.

At present, a convenient and reliable biomarker for the prediction of anti-PD-1 response in HCC is still lacking. However, some evidence showed that the change of AFP level was corelated to anti-PD-1 response. We previously found that patients with AFP > 10 ng/dL before treatment and declined > 10% within 4 weeks could predict image response [16]. Shao et al. used a 20% decline as the cut-off level of the AFP response [17]. In this study, we found that the response of AFP on day 14 can early predict the subsequent image response. This may suggest that anti-PD-1 can exert its anti-tumor effect at such an early stage. Furthermore, we found that the more AFP level declined, the deeper tumor response. Patients with AFP declined > 66% on day 28, the CR rate could even reach 58%. Conversely, when AFP declined less than 10%, the ORR was only 3%. These further addressed the important negative predictive value of AFP response. Taken together, the change of AFP value is parallel to that of the image response.

Grade 3/4 irAE was about 25% with nivolumab and pembrolizumab alone, but it rose to 29–53% with the combination of nivolumab and ipilimumab [18], 56% with atezolizumab and bevacizumab [6], and 73% with pembrolizumab and lenvatinib [19]. In our study, grade 3/4 irAE was only 10.3% with the combination of anti-PD-1 and sorafenib. How to strike a balance between efficacy and toxicity remained an important issue at present. Furthermore, the median time to onset of high grade irAE was 20 days in this study, which is compatible with a previous report that showed fatal toxic effects typically occurred early (14.5 days) after immunotherapy initiation [20]. Therefore, caution is advised during this period when the risk of fetal irAE is high but the efficacy can’t be estimated yet.

Regarding organ-specific tumor response, lung and peritoneal metastasis have the highest response rates, followed by liver metastasis in a melanoma study [21]. In HCC, Lu et al. published a similar result, which showed the highest response rates were the lungs (41.2%) and intra-abdomen (38.9%), followed by the lymph node (26.3%) and liver (22.4%) (28). Our study showed that the metastatic organ of the lungs (33.4%), peritoneum (40.0%) and adrenal glands (50.0%) had high response rate, followed by the liver (19.4%) and lymph node (14.3%). Conversely, the response of bone metastasis and tumor thrombosis was quite low (< 10%). Such consistency implies that the tumor microenvironment of various organs may potentially influence the therapeutic effect.

This study has several limitations. First, this is a retrospective study, so selection bias is inevitable. With the use of propensity-score matching analysis, we minimized the bias between groups. Second, the mean of sorafenib starting doses in this study was about half of the recommended dose, which may underestimate the efficacy of combination therapy. However, the optimal escalation strategy of sorafenib is controversial [22]. The adequate dose of multi-kinase inhibitors to combine with anti-PD-1 is also unclear. Therefore, this study provided an important reference for such combination. Finally, to avoid recall bias for low grade AE, this article only analyzed grade 3/4 AE.

## Conclusions

This propensity-score matching study showed that the combination of anti-PD-1 and sorafenib had better tumor control and prolonged PFS and OS with tolerated toxic profile in advanced HCC. However, a well-designed prospective randomized study is needed to confirm this finding.

## Supplementary Information

**Additional file 1 **: **Supplemental Table 1**. Grade 3/4 adverse event.

**Additional file 2.**

## Data Availability

Data available on request from the authors.

## Abbreviations

VEGF: Vascular endothelial growth factor
HCC: Hepatocellular carcinoma
MKI: Multiple-kinase inhibitors
BCLC: Barcelona clinic liver cancer
PFS: Progression-free survival
ToR: Time to response
DoR: Duration of response
OS: Overall survival
PVT: Portal vein thrombosis
