# Supplementary material for: Anti-PD-1 combined sorafenib versus anti-PD-1 alone in the treatment of advanced hepatocellular cell carcinoma: a propensity score-matching study
Source: BMC Cancer. 2022 Jan 11;22:55. doi: 10.1186/s12885-022-09173-4 (PMC8753926; doi:10.1186/s12885-022-09173-4)
Supplement: Supplementary file 1 — Additional file 1 : Supplemental Table 1. Grade 3/4 adverse event. [file 12885_2022_9173_MOESM1_ESM.docx]

### Supplemental table 1. Grade 3/4 adverse event

|  | Anti-PD-1 plus sorafenib (n=58) | | Anti-PD-1 alone (n=42) | | *p* value |
| --- | --- | --- | --- | --- | --- |
| **Grade 3/4 AE** | 6 | 10.3% | 3 | 7.1% | 0.73 |
| Hepatitis | 1 | 1.7% | 1 | 2.4% |  |
| Pneumonitis | 1 | 1.7% | 1 | 2.4% |  |
| Skin toxicity† | 2 | 3.4% | 1 | 2.4% |  |
| Cholangitis | 1 | 1.7% | 0 | 0.0% |  |
| Sick-sinus syndrome | 1 | 1.7% | 0 | 0.0% |  |

irAE, immune-related adverse event;

†Including one toxic epidermal necrolysis and one psoriasis flare-up in combination group
